# The Misapplication of Gold Crowns in Bridgework

**Published:** 1897-11

**Authors:** W. W. H. Thackston

**Affiliations:** Farmville, Va.


### The Misapplication of Gold Crowns in Bridgework.

BY DR. W. W. H. THACKSTON, FARMVILLE, VA.

Abstract of a paper read before the Southern Dental Association, Old Point Comfort, Va., August, 1897.

After reviewing in scathing terms the degradation of prosthetic dentistry by the "Cheap Johns" after the advent of rubber and the base metals, until it became a question among the better class of honorable men in the profession of sloughing off the prosthetic department altogether and leaving it in the hands of the men who had succeeded in almost entirely monopolizing the patronage of the people who became infatuated with the idea of cheap dentistry, Dr. Thackston drew an equally vivid picture of the more recent revival and the reinstatement of gold and platinum, and the introduction of comparatively new operations of crowning with gold worn, decayed and broken down stumps of natural teeth and the substitution of "Bridges" for the more cumbrous plates.

With correctly made and judiciously applied crowns and bridges, and with an imperishable *plastic* tooth-filling, stable, non-shrinkable, inexpensive, and of shades and colors matching the natural teeth, the very perfection and ideal of prosthetic and operative dentistry would be realized.

But alas! We have not yet discovered the unchangeable "plastic" and the misapplication of crowns and bridges, their faulty construction and equally faulty adjustment disappoint and disgust our patients and create distrust of our resources to meet their wants and fulfill our promises of ability and comfort. We are witnesses to too many gross violations of all hygienic and asthetic rules and requirements.

The safe and sure maxims for our prosthetic brotherhood, are as follows:

*First*—A sound and healthy condition of the hard and soft tissues of the mouth, including the gums, membranes, teeth, and roots of teeth.

*Second*—Porcelain "crowns" and "facings" for all anterior and exposed teeth.

*Third*—Perfect adaptation and artistic finish for each and every piece designed for insertion in the human mouth.

